# Big city, small world: density, contact rates, and transmission of dengue across Pakistan

**DOI:** 10.1098/rsif.2015.0468

**Published:** 2015-10-06

**Authors:** M. U. G. Kraemer, T. A. Perkins, D. A. T. Cummings, R. Zakar, S. I. Hay, D. L. Smith, R. C. Reiner

**Affiliations:** 1 Department of Zoology, University of Oxford, Oxford OX1 3PS, UK; 2 Department of Biological Sciences and Eck Institute for Global Health, University of Notre Dame, Notre Dame, IN 46556, USA; 3 Fogarty International Center, National Institutes of Health, Bethesda, MD 20892, USA; 4 Department of Epidemiology, Johns Hopkins University, Bloomberg School of Public Health, Baltimore, MD 21205, USA; 5 Department of Public Health, University of Punjab, Lahore 54590, Pakistan; 6 Wellcome Trust Centre for Human Genetics, University of Oxford, Oxford OX3 7BN, UK; 7 Institute for Health Metrics and Evaluation, University of Washington, Seattle, WA 98121, USA; 8 Sanaria Institute for Global Health and Tropical Medicine, Rockville, MD 20850, USA; 9 Department of Epidemiology and Biostatistics, Indiana University School of Public Health, Bloomington, IN 47405, USA

**Keywords:** dengue, epidemiology, heterogeneity, mixing, spatial dynamics, spatial accessibility

## Abstract

Macroscopic descriptions of populations commonly assume that encounters between individuals are well mixed; i.e. each individual has an equal chance of coming into contact with any other individual. Relaxing this assumption can be challenging though, due to the difficulty of acquiring detailed knowledge about the non-random nature of encounters. Here, we fitted a mathematical model of dengue virus transmission to spatial time-series data from Pakistan and compared maximum-likelihood estimates of ‘mixing parameters’ when disaggregating data across an urban–rural gradient. We show that dynamics across this gradient are subject not only to differing transmission intensities but also to differing strengths of nonlinearity due to differences in mixing. Accounting for differences in mobility by incorporating two fine-scale, density-dependent covariate layers eliminates differences in mixing but results in a doubling of the estimated transmission potential of the large urban district of Lahore. We furthermore show that neglecting spatial variation in mixing can lead to substantial underestimates of the level of effort needed to control a pathogen with vaccines or other interventions. We complement this analysis with estimates of the relationships between dengue transmission intensity and other putative environmental drivers thereof.

## 1. Introduction

The transmission dynamics of all infectious diseases depend on a few basic but key determinants: the availability of susceptible and infectious hosts, contacts between them and the potential for transmission upon contact. Susceptibility is shaped primarily by historical patterns of transmission, the natural history of the pathogen, the host's immune response and host demography [1]. What constitutes an epidemiologically significant contact depends on the pathogen's mode of transmission [2], and structure in contact patterns can be influenced by transportation networks and the spatial scale of transmission [3], by host heterogeneities such as age [4], and dynamically in response to the pathogen's influence on host behaviour [5]. Whether transmission actually occurs during contact between susceptible and infectious hosts often depends heavily on environmental conditions [6–8]. Disentangling the relative roles of these factors in driving patterns of disease incidence and prevalence is a difficult but central pursuit in infectious disease epidemiology, and mathematical models that capture the biology of how these mechanisms interact represent an indispensible tool in this pursuit [9].

The time-series susceptible–infected–recovered (TSIR) model was developed by Finkenstädt & Grenfell [10] to offer an accurate and straightforward way to statistically connect mechanistic models of infectious disease transmission with time-series data. Among other features, TSIR models readily account for inhomogeneous mixing in a phenomenological way by allowing for rates of contact between susceptible and infectious hosts to depend on their densities nonlinearly. Although this is a simple feature that can be incorporated into any model based on mass-action assumptions—indeed, earlier applications pertained to inhomogeneous mixing in predator–prey systems [11]—the ‘mixing parameters’ that determine the extent of this nonlinearity have primarily been fitted to empirical data in applications of the TSIR model to measles, cholera, rubella and dengue [12–15]. Applied to discrete-time models such as the TSIR, mixing parameters also have an interpretation as corrections for approximating a truly continuous-time process with a discrete-time model [16,17]. In no application of the TSIR model to date has the potential for variation in these parameters been assessed, leaving the extent to which inhomogeneity of mixing varies across space and time as an open question in the study of infectious disease dynamics.

There are a number of reasons why mixing might vary in time or space. Seasonal variation in mixing might arise because of travel associated with labour [18], religious events [19] or vacation [20], or because of the timing of school openings in the case of influenza [21]. Spatial variation in mixing could arise because of cultural differences at geographical scales [3,22,23], because of variation in the density and quality of roads [24], or because of variation in human densities and myriad-associated factors [13,25]. For vector-borne diseases, variation in mixing is amplified even further by variation in vector densities [26], which effectively mediate contact between susceptible and infectious hosts.

Dengue is a mosquito-borne viral disease with a strong potential for spatial variation in mixing [27,28]. The dominant vectors of dengue viruses (*Aedes* spp.) thrive in areas where they are able to associate with humans, as humans provide not only a preferred source of blood but also water containers that the mosquitoes use for egg laying and for larval and pupal development [29]. Two additional aspects of *Aedes* ecology—limited dispersal distance of the mosquito [30] and daytime biting [31]—imply that human movement should be the primary means by which the viruses spread spatially [2]. Indeed, analyses of dengue transmission dynamics at a variety of scales have strongly supported this hypothesis [32–35]. To the extent that human movement in dense urban environments is more well mixed than elsewhere, there are likely to be differences in the extent of inhomogeneous mixing in peri-urban and rural areas. This is also presumably the case for directly transmitted pathogens, but with a potentially even stronger discrepancy for dengue due to the urban–rural gradient in mosquito densities.

To assess the potential for spatial variation in the inhomogeneity of mixing as it pertains dengue transmission, we performed an analysis of district-level time series of dengue transmission in the Punjab province of Pakistan using a TSIR model with separate mixing parameters for urban and rural districts. We likewise made estimates of the relationships between density-independent transmission potential and putative drivers thereof, such as temperature, to allow for the relative roles of extrinsic and intrinsic factors to be teased apart. Finally, we performed mathematical analyses of the fitted model to assess the significance of spatial variation in mixing inhomogeneity for how time-series data are interpreted and used to guide control efforts.

## 2. Material and methods

We obtained daily dengue case data aggregated at hospital level from Punjab province provided by the Health Department Punjab, Pakistan, between 2011 and 2014. In total, 47 156 suspected and confirmed dengue cases were reported in 109 hospitals. All hospitals were subsequently geo-located using ‘Google maps’ (http://www.maps.google.com) similar to methods described here [36]. Hospitals that could not be identified were removed from the database. The hospitals were then assigned to a district within Punjab, Pakistan by their spatial location. A total of 21 182 cases alone were reported from the year 2011, which affected almost the entire province. Many more cases occurred in Lahore (35 348) compared to all other districts (8808) (table 1). A breakdown per year and each province is provided in electronic supplementary material, table S1, and additional information about collection can be found in the electronic supplementary material, appendix. No information on dengue serotypes were available. However, the predominant serotype circulating in Punjab province, Pakistan is that of DENV-2 [37].

**Table 1.** Reported cases by year in Lahore and all other districts.

	2011	2012	2013	2014	total
Lahore	18 020	4013	11 516	1799	35 348
other	3162	500	2356	2790	8808

### 2.1. Covariate selection and processing

**Figure 1.**
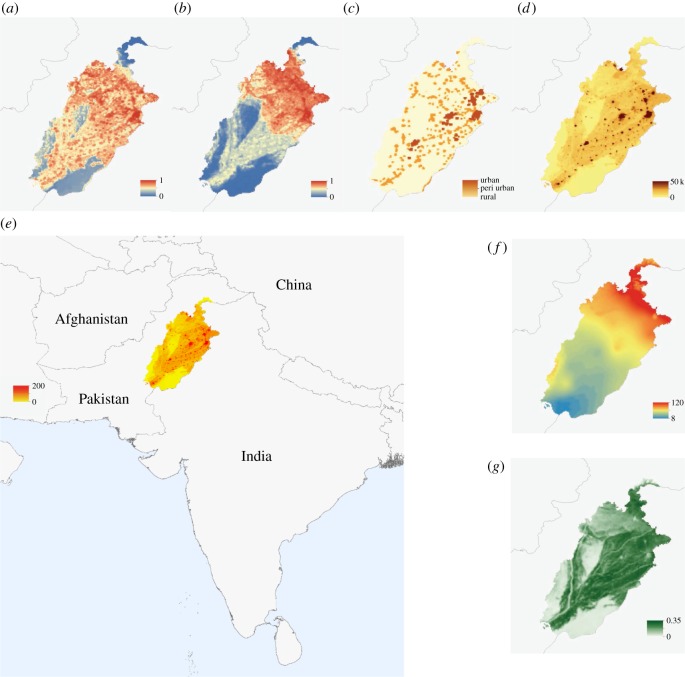
Covariates used in this study to derive environmental drivers of transmission. *Aedes aegypti* probability of occurrence (*a*); *A. albopictus* probability of occurrence (*b*); urbanicity (*c*); weighted urban accessibility (*d*); population density and study area (*e*); precipitation (*f*); EVI mean (*g*).

Environmental conditions are instrumental in defining the risk of transmission of dengue [28]. Transmission is limited by the availability of a competent disease vector. Due to a lack of resources and political instability, no comprehensive nationwide entomological surveys have been performed in Pakistan. Therefore, we use a probabilistic model to infer the probability of occurrence of *Aedes aegypti* and *Aedes albopictus* in Pakistan derived from a globally comprehensive dataset containing more than 20 000 records for each species (figure 1*a*,*b*). In short, a boosted regression tree model was applied that predicts a continuous spatial surface of mosquito occurrence from a fitted relationship between the distribution of these mosquitoes and their environmental drivers. A detailed description of the occurrence database and modelling outputs are available here [36,38,39]. Such model outputs have proved useful in identifying areas of risk of transmission of dengue as well as malaria [28,40,41]. Other important environmental conditions defining the risk of transmission of dengue are temperature, water availability and vegetation cover [42]. To account for such variation, raster layers of daytime land surface temperature were processed from the MOD11A2 satellite, gap-filled to remove missing values, and then averaged to a monthly temporal resolution for all 4 years [43]. The density of vegetation coverage has been shown to be associated with vector abundance [44]. Moreover, vegetation indices are useful proxies for precipitation and may be used to derive the presence of standing water buckets that are habitats for the *Aedes* mosquitos [45]. The same method was again applied to derive the enhanced vegetation index (EVI) from the MOD11A2 satellite to produce 16-day and monthly pixel-based estimates for 2011–2014 (figure 1*g*) [46]. Due to the inherent delay between rainfall and daily temperature influencing mosquito population dynamics and those mosquitoes contributing to an increase in DENV transmission, we consider both, the influence of the current temperature, vegetation indices and precipitation, data on current transmission as well as the values of those covariates the time step before (figure 1*f*).

We used population count estimates on a 100 m resolution that were subsequently aggregated up to match all other raster layers to a 5 × 5 km resolution for the year 2015 (http://www.worldpop.org) (figure 1*e*). In a follow-up analysis to our primary investigation into the climatological drivers of dengue transmission, we included several density-based covariates. We derived a weighted accessibility metric that includes both, population density and urban accessibility, a metric commonly used to derive relative movement patterns [24,47]. This map shows a friction surface, i.e. the time needed to travel through a specific pixel (figure 1*d*). We also used an urban, peri-urban and rural classification scheme to quantify patterns of urbanicity based on a globally available grid [48] (figure 1*c*). All covariates and case data were aggregated and averaged (where appropriate) to a district level.

### 2.2. Model

Following Finkenstädt & Grenfell [10], we assume a general transmission model of2.1
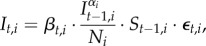
where *I_t_*_,*i*_ is the number of infected and infectious individuals and *S_t_*_,*i*_ the number of susceptible individuals, at time *t* in district *i*, *N_i_* is the population of district *i*, and *β_t_*_,*i*_ is the covariate driven contact rate. We assume each individual to be susceptible as the 2011 epidemic is the first large dengue outbreak. The mixing parameter for the *i*th district is given by *α_i_*; when *α_i_* is equal to 1, the population has homogeneous mixing where values less than one can either indicate inhomogeneous mixing or a need to correct for the discretization of the continuous-time process. *β_t_*,*_i_* was fitted using covariates shown in figure 1. Finally, the error terms 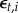 are assumed to be independent and identically log-normally distributed random variables.

### 2.3. Model selection

The term *β_t,i_* is fit using generalized additive model regression [49–51]. The time-varying climatological covariates are all fit as a smooth spline, while all other covariates enter *β_t,i_* linearly. For example, if covariate *X*_1_ and *X*_2_ are time varying and *X*_3_ and *X*_4_ are temporally constant, then we fit *β* as2.2
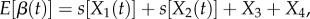
where *s* are smooths.

Additionally, unexplained seasonal variation is accounted for using a 12-month periodic smooth spline.

Model selection was performed using backwards selection. Two base models were investigated. First, a climate-only model was created using only the climatological and environmental suitability covariates. Second, a ‘full’ model using the density-dependent covariates as well as the climatological covariates were combined into a single model which was then subjected to backwards selection. For both models, the mixing coefficient was initially set equal for each district and once a final model was arrived upon, the mixing coefficient for Lahore was allowed to vary separately from the other coefficients. All model fitting was conducted using R [52] and the ‘mgcv’ package [51]. Models are fit by maximizing the restricted maximum likelihood [53] to reduce bias and over-fitting of the smooth splines. The model source code and processing of covariates will be made available in line with previous projects [54].

### 2.4. Model analysis

To explore the potential significance of spatial variation in mixing parameters, we conducted an analysis to probe the inherent mathematical trade-off between the mixing parameter *α* and the density-independent transmission coefficient *β*. Specifically, to answer the question, what difference in local transmission would be necessary to account for a difference in mixing while achieving identical transmission dynamics. To explore this, we used equation (2.1) to establish:2.3
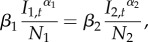
from which we obtained2.4
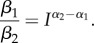


We then examined how variation in *l* and *α*_2_ − *α*_1_ affected the left-hand side of equation (2.4) and likewise the critical proportion of the population to control in order to effect pathogen elimination, which, under our model, is *p*_c_ = 1 − (1/*β*).

## 3. Results

### 3.1. Description of case distribution

The majority of cases were clustered in Lahore, the capital of Punjab province. Ongoing transmission appeared to be focal in three (Vehari, Rawalpindi and Lahore) districts and to have spread through infective ‘sparks’ to smaller more rural provinces.

### 3.2. Model selection

**Figure 2.**
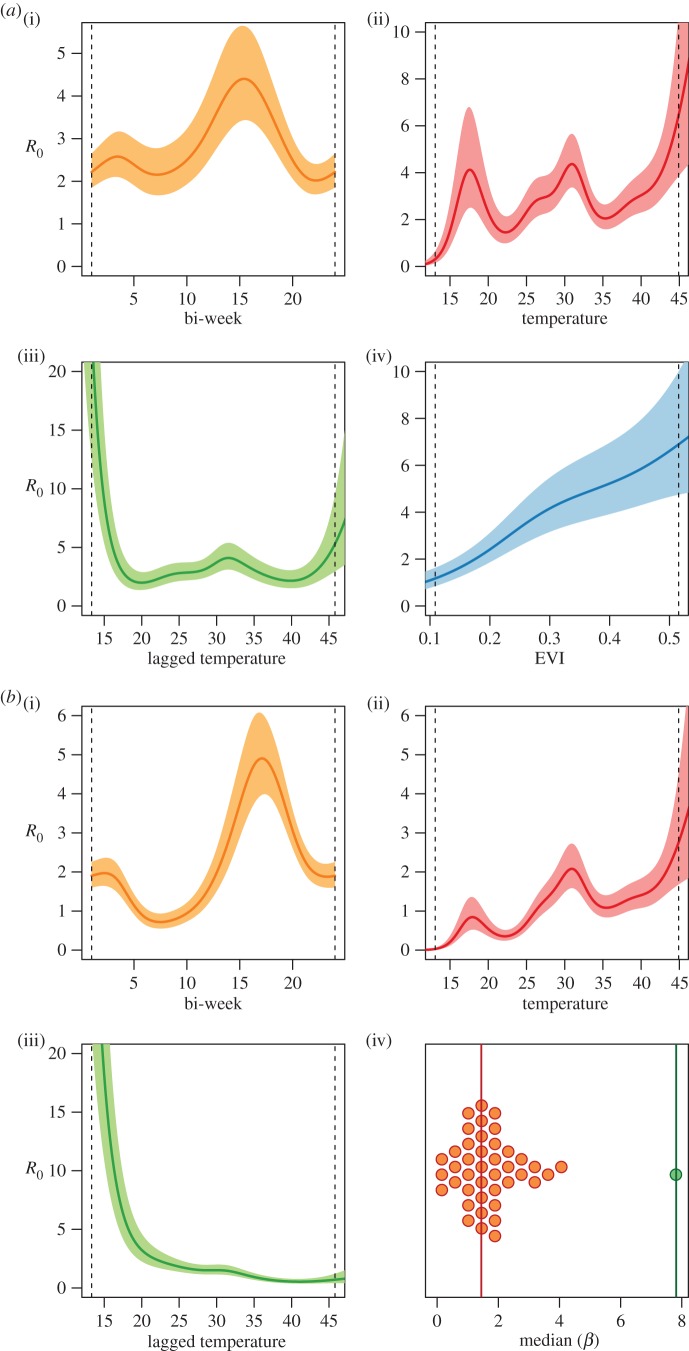
Model outputs using a backwards model selection procedure in the model using climatological variables (*a*, i–iv), and including the density-dependent variables (*b*, i–iv). Every subplot shows the predictions of the model for the indicated parameter carrying across the indicated range and every other parameter set to their mean. Figure (*b*, iv) shows the differences in the transmission coefficient from Lahore (green) and all other districts (red).

**Figure 3.**
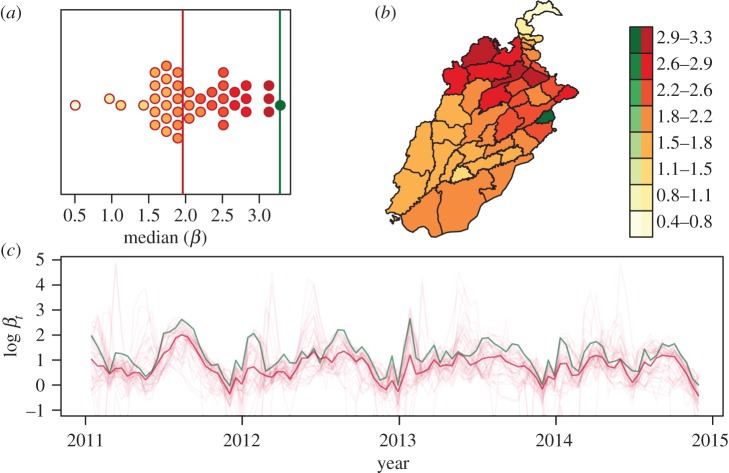
Average distribution of *R*_0_ with green representing Lahore versus red all other districts (*a*), their geographical distribution (*b*) and over time in which the green line again is representing Lahore versus the red line representing all other districts (*c*). Outputs shown here correspond to the model without including density-related variables.

To disentangle the different aspects of dengue dynamics and their drivers, we used a model containing only the climatological covariates and performed backwards model selection until each covariate in the model was significant at a 5% level. This resulted in a model that explained 76.9% of the deviance and that had an adjusted *R*^2^ of 0.746. Among the yearly averaged covariates, EVI and precipitation remained in the model, as well as the derived *A. albopictus* range map (*p* = 8.21 × 10^−4^, 0.01, and 3.9 × 10^−5^, respectively). Interestingly, when we substituted the derived *A. aegypti* map for the *A. albopictus* map, the deviance explained changed very little to 76.8%. For climatological covariates that were fit as smooth splines, temperature, lagged temperature and EVI remained in the model (figure 2, *p*-value of 0.010, 0.030 and 0.030 with effective degrees of freedom 7.55, 5.47 and 1.83, respectively). There was a significant amount of periodic variation unexplained by the climatological covariates alone, as the ‘seasonality’ covariate was retained by the model selection algorithm (figure 2, *p* = 0.0034). The estimated median values for *R*_0_ per district were clustered around 2 (mean = 2.1), and their geographical distribution indicated a clear trend towards districts with larger populations (figure 3). Finally, the mixing coefficient was significantly lower than 1 (*α* = 0.69, 95% CI = (0.614, 0.771), *p* = 1.6 × 10^−14^).

To understand these differences, the final model was then compared to a nested model in which the coefficient for Lahore was allowed to vary independently of all other districts. Deviance explained increased to 77% and adjusted *R*^2^ increased to 0.753. Further, the mixing coefficient for Lahore (*α* = 0.74) was significantly larger than the mixing coefficient for the other districts (*α* = 0.59, *p* = 0.0068) (electronic supplementary material, figure S1). The median *R*_0_ for Lahore was estimated at 3.28, the highest among all districts.

To assess the extent to which the variation in mixing coefficients could be explained by other covariates, we considered the possibility that movement accounted for the differences in the mixing coefficients between Lahore and the other districts. The density-dependent covariates (described earlier) were then added to the full model and backwards selection was performed again. The resulting model explained 78.6% of the deviance, had an adjusted *R*^2^ of 0.763 and was superior to the final climatological model based on AIC (699.23 versus 714.83). Yearly averaged EVI, normalized difference vegetation index and precipitation were all significant (*p* = 8.7 × 10^−5^, 0.00024 and 0.00028, respectively). Again, the derived *A. albopictus* map was significant (*p* = 0.00816). For climatological covariates fit as smooth splines, only temperature and lagged temperature were found to be significant (figure 2*b*, *p* = 4.0 × 10^−5^ and 0.0013, and effective degrees of freedom 7.61 and 4.81, respectively), and there was still a significant ‘seasonality’ effect (figure 2*b*, *p* = 4.0 × 10^−7^, effective degrees of freedom 4.48). The best-fit mixing coefficient was *α* = 0.58, barely lower than the mixing coefficient for non-Lahore districts in the climatological model. The estimated median *R*_0_ again clustered around 2 (mean = 1.8), and again the *R*_0_ for Lahore was largest, but in this model it was considerably larger than in the climatological model (Lahore *R*_0_ = 7.82, figure 2*b*). Full details of the best-fit model parameters are shown in electronic supplementary material, table S2–S5.

Two of the density-dependent covariates remained in the model: the urban map (*p* = 0.01) and the weighted access map (*p* = 3 × 10^−5^). When the nested model that allowed Lahore's mixing coefficient to vary was fitted, there was no significant difference between the two mixing coefficients (*p* ≈ 1).

### 3.3. Model analysis

**Figure 4.**
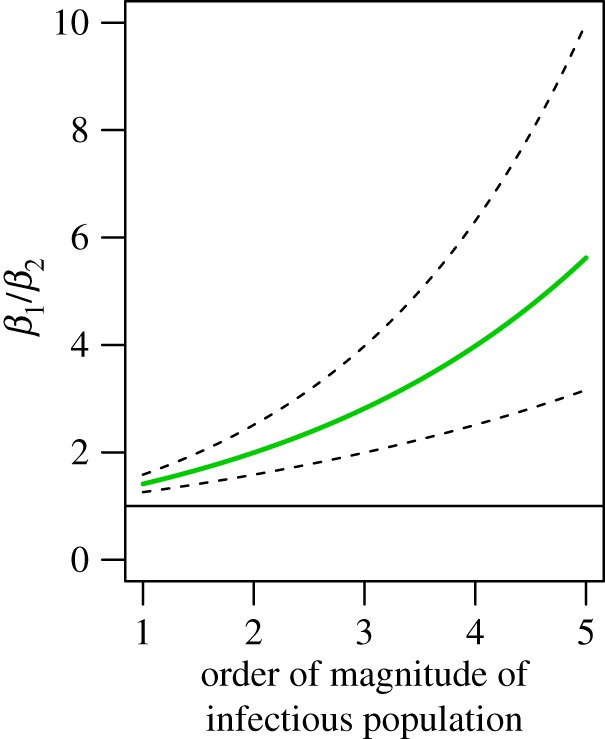
Ratio of betas (*R*_0_) assuming equal force of infection and a difference in *α*_2_–*α*_1_ of 0.2, 0.15 (green), and 0.1, from top to bottom. The straight line indicates a ratio of 1. Order of magnitude of the infectious population refers to number of infectious people in powers of 10; i.e. the range is 10–100 000.

**Figure 5.**
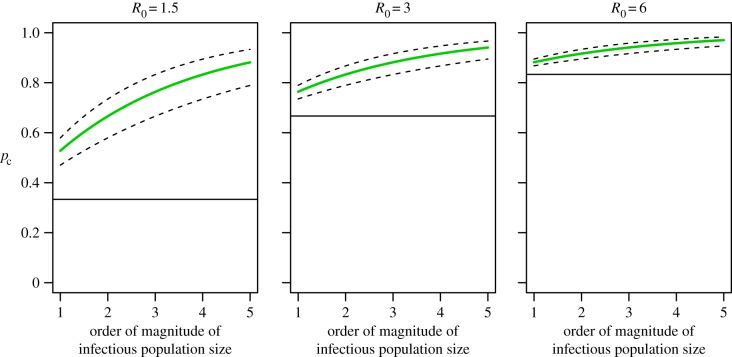
Critical proportion of the population to control in population 2 as a function of *R*_0_ in population 1, the order of magnitude of the infectious numbers in each population, and a difference in *α*_2_–*α*_1_ of 0.1, 0.15 (green) and 0.2. The straight line indicates the critical proportion assuming the *α* in each population are equal. Order of magnitude of the infectious population refers to number of infectious people in powers of 10; i.e. the range is 10–100 000.

Given a difference in estimates of the mixing parameters between Lahore and elsewhere of 0.15, we analysed equation (2.3) to assess the bias in estimates of the transmission coefficient that would result from ignoring this extent of variation in the inhomogeneity of mixing displayed between two areas. For the purpose of *ceteris paribus* comparisons, we assumed equal force of infection but varied it across several orders of magnitude. Depending on the order of magnitude, estimates of transmission coefficients made if overestimating the mixing parameter by 0.15 could easily result in a two- to threefold underestimate in the transmission coefficient (figure 4). For realistic ranges of the transmission coefficient for dengue, and equivalently for the basic reproductive number *R*_0_ under the present model, this extent of underestimation of *R*_0_ could lead to underestimating the critical proportion of the population to which vaccines or other interventions must be applied by 20–30% (figure 5).

## 4. Discussion

Our results point to considerable spatial heterogeneity in the inhomogeneity of mixing and the strength of an associated nonlinearity in transmission along an urban–rural gradient. This regional variability in mixing has direct implications for estimates of the basic reproductive number of dengue in our study region and elsewhere. Although the potential for such bias in estimates of the basic reproductive number has been shown in a theoretical context [26,55], we provide quantitative estimates of the extent of this problem by interfacing models with a rich spatio-temporal dataset. Our results have implications for estimates of population-level parameters not only for dengue but also for other infectious diseases [13,56–60] and possibly even more broadly in ecology [11].

Our analysis revealed significant differences in the inhomogeneity of mixing between urban and rural settings and found that a population-weighted urban accessibility metric was able to account for differences in mixing between these settings. Mixing is presumably influenced directly by human behaviour and has been shown to be highly unpredictable, largely dependent on the local context and the spatial and temporal scale [61]. In this study, however, we could show that the density-dependent covariate we considered was able to capture the influence of these behavioural effects on a district level. Once differences in the inhomogeneity of mixing were accounted for, estimated *R*_0_ values indicated considerably larger differences between transmission potential in Lahore and all other districts. Synchronizing more accurate geo-referenced data would allow for the assessment of the extent to which the relationship between ‘mixing parameters’ and urban accessibility is dependent on the spatial scale at which data are aggregated [26,62]. In the case of dengue, this has been limited specifically by the availability of high-resolution data [63]. Complementing such an analysis with measurement of social contact patterns could be important for exploring this relationship in even more detail [22,64,65] and could be informed by mathematical models that explored this relationship previously for other diseases [13,66]. Another encouraging result from our analysis was the finding that large-scale mosquito suitability surfaces helped capture the environmental determinants of dengue transmission [28].

Intervention strategies are contingent on both understanding key environmental drivers of transmission and the dynamics of ongoing human-to-human transmission, particularly in outbreak situations [67]. Environmental drivers such as seasonal fluctuations in rainfall, temperature, vegetation coverage or mosquito abundance will help guide surveillance and control efforts targeted mostly towards the mosquito vector and its ecology [68]. Once infection occurs, an important and unresolved question for dengue is how to best optimize the delivery of intervention strategies to reduce disease incidence, which is largely determined by *R*_0_. Our analysis shows that the interaction between mixing parameters and force of infection has potentially large implications for optimizing targeted intervention, particularly in countries where transmission is high and resources are scarce [69]. In fact, this may be even more important in areas of low transmission where incidence appears to be more focal [70]. Again, however, more attention is needed to determine the spatial and temporal resolution of appropriate intervention strategies and the effects of key covariates and model parameters [62]. Empirical understanding of the spatial scale that is most appropriate for carrying out large-scale interventions remains unknown.

Once transmission has occurred in one place, understanding not only spatial heterogeneity in transmission dynamics but also their subsequent spread in mechanistic stochastic models would help to empirically determine the propagation of the disease [71]. Interest in spatial spread dynamics has risen with increasing importation of dengue into heretofore non-endemic areas due to travel and trade continentally and internationally [72]. Exploration of the case data in Pakistan that we analysed here suggests that the virus spreads along major transport routes from Lahore to Karachi and north to Rawalpindi. Using results presented here on mixing coefficients and environmental drivers will help pinpoint areas of major risk of importation more accurately, especially in the case of recurring epidemics. We explored the consequences of a spatially differentiated mixing coefficient in the context of transmission potential within this analysis. Using the fitted relationships of the environmental drivers of transmission and *R*_0_ will enable future analyses and comparisons between diseases and geographical regions. In this context, it will be instrumental to integrate a variety of movement and social network models with the evidence presented here to infer more accurately how the geographical spread of dengue is determined.

## Supplementary Material

Electronic supplementary material.doc
