# Supplementary material for: Big city, small world: density, contact rates, and transmission of dengue across Pakistan
Source: J R Soc Interface. 2015 Oct 6;12(111):20150468. doi: 10.1098/rsif.2015.0468 (PMC4614486; doi:10.1098/rsif.2015.0468)
Supplement: Electronic supplementary material.doc [file rsif20150468supp1.doc]

**Electronic supplementary material**

**Big city, small world: Density, contact rates,**

**and transmission of dengue across Pakistan**

Kraemer, M.U.G.*^1^, Perkins, T. A.^2,3^, Cummings, D.A.T.^4^, Zakar, R.^5^, Hay, S.I.^3,6,7^, Smith, D.L.^1,3,8^, and Reiner, R.C.*^3,9^

1. Department of Zoology, University of Oxford, Oxford, OX1 3PS, United Kingdom;
2. Department of Biological Sciences and Eck Institute for Global Health, University of Notre Dame, Notre Dame, IN 46556, United States;
3. Fogarty International Center, National Institutes of Health, Bethesda, MD 20892, United States;
4. Department of Epidemiology, Johns Hopkins University, Bloomberg School of Public Health, Baltimore, MD 21205, United States;
5. Department of Public Health, University of Punjab, Lahore, 54590, Pakistan;
6. Wellcome Trust Centre for Human Genetics, University of Oxford, Oxford, OX3 7BN, United Kingdom;
7. Institute for Health Metrics and Evaluation, University of Washington, Seattle, WA 98121, United States;
8. Sanaria Institute for Global Health and Tropical Medicine, Rockville, MD 20850, United States;
9. Department of Epidemiology and Biostatistics, Indiana University School of Public Health, Bloomington, IN 47405, United States;

**Table S1: Reported case numbers per year for each district of the study region, Punjab Province, Pakistan.**

**
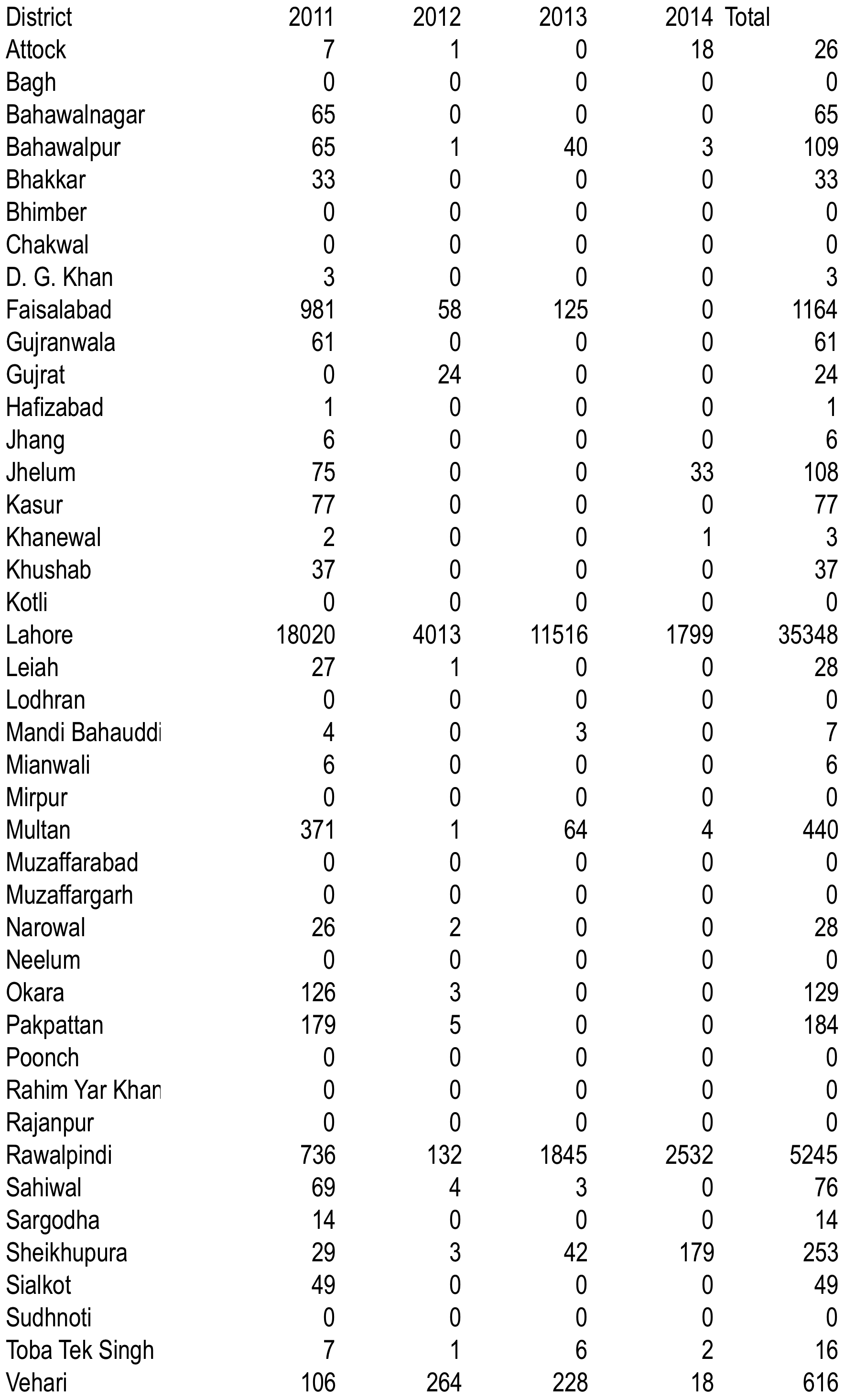
**

| Term | Estimate | Std. Error | t-value | p-value |
| --- | --- | --- | --- | --- |
| Intercept | **2.51** | **0.679** | **3.696** | **0.00028** |
| 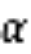 | **0.690** | **0.040** | **17.288** | **< 2e-16** |
| EVI  (yearly average) | **-8.78** | **2.499** | **-3.392** | **0.00082** |
| *Ae. albopictus* | **2.28** | **0.544** | **4.196** | **3.93e-5** |
| Precipitation (yearly average) | **-0.021** | **0.0079** | **-2.590** | **0.0102** |
| Term | **edf** | **Ref. df** | **F** | **p-value** |
| “Seasonality” | **3.44** | **8.00** | **1.566** | **0.0034** |
| Temperature | **7.55** | **8.47** | **2.537** | **0.0102** |
| 2-week lagged temperature | **5.47** | **6.67** | **2.300** | **0.0304** |
| EVI | **1.83** | **2.33** | **3.373** | **0.0299** |

**Table S2: Environmental model: no variation in**
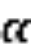
**.**

**Table S3: Environmental model:
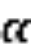
** in Lahore differs from other districts.

| Term | Estimate | Std. Error | t-value | p-value |
| --- | --- | --- | --- | --- |
| Intercept | **2.548** | **0.676** | **3.769** | **0.0002** |
| 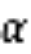 (Lahore) | **0.741** | **0.043** | **17.088** | **<2e-16** |
| 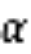 (Not Lahore) | **0.594** | **0.055** | **10.887** | **<2e-16** |
| EVI  (yearly average) | **-7.636** | **2.527** | **-3.022** | **0.003** |
| *Ae. albopictus* | **1.159** | **0.679** | **1.708** | **0.089** |
| Precipitation (yearly average) | **-0.008** | **0.009** | **-0.870** | **0.385** |
| Term | **edf** | **Ref. df** | **F** | **p-value** |
| “Seasonality” | **3.238** | **8.000** | **1.278** | **0.0095** |
| Temperature | **7.502** | **8.435** | **3.195** | **0.0016** |
| 2-week lagged temperature | **5.866** | **7.067** | **2.371** | **0.0231** |
| EVI | **2.179** | **2.784** | **3.690** | **0.0154** |

**Table S4: Full model: no variation in
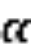
**.

| Term | Estimate | Std. Error | t-value | p-value |
| --- | --- | --- | --- | --- |
| Intercept | **-34.83** | **9.803** | **-3.553** | **0.00047** |
| 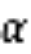 | **0.578** | **0.0451** | **12.824** | **<2e-16** |
| EVI  (yearly average) | **-60.12** | **15.04** | **-3.998** | **8.71e-5** |
| NDVI  (yearly average) | **0.0383** | **0.0103** | **3.737** | **0.00024** |
| *Ae. albopictus* | **2.745** | **1.028** | **2.669** | **0.00826** |
| Weighted Access | **2.742e-5** | **6.464e-6** | **4.242** | **3.26e-5** |
| Urbanicity | **-2.468** | **0.9494** | **-2.600** | **0.00776** |
| Precipitation (yearly average) | **-0.0684** | **0.0185** | **-3.697** | **0.00028** |
| Term | **edf** | **Ref. df** | **F** | **p-value** |
| “Seasonality” | **4.480** | **8.000** | **4.173** | **3.97e-7** |
| Temperature | **7.607** | **8.504** | **4.396** | **4.14e-5** |
| 2-week lagged temperature | **4.823** | **5.981** | **3.793** | **0.0013** |

**Table S5: Full model:**
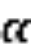
 **in Lahore differs from other districts.**

| Term | Estimate | Std. Error | t-value | p-value |
| --- | --- | --- | --- | --- |
| Intercept | **-35.92** | **9.93** | **-3.618** | **0.00037** |
| 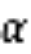 (Lahore) | **0.612** | **0.0643** | **8.510** | **<2e-16** |
| 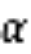 (Not Lahore) | **0.555** | **0.0566** | **9.802** | **<2e-16** |
| EVI  (yearly average) | **-61.59** | **15.19** | **-4.055** | **6.95e-5** |
| NDVI  (yearly average) | **0.0395** | **0.010** | **3.8** | **0.00019** |
| *Ae. albopictus* | **2.81** | **1.033** | **2.721** | **0.0070** |
| Weighted Access | **2.42e-5** | **7.86e-6** | **3.072** | **0.0024** |
| Urbanicity | **-2.33** | **0.9697** | **-2.403** | **0.0171** |
| Precipitation (yearly average) | **-0.069** | **0.0186** | **-3.733** | **0.00024** |
| Term | **edf** | **Ref. df** | **F** | **p-value** |
| “Seasonality” | **4.350** | **8.000** | **3.817** | **1.31e-6** |
| Temperature | **7.620** | **8.511** | **4.335** | **4.97e-5** |
| 2-week lagged temperature | **4.764** | **5.916** | **3.680** | **0.00178** |

**Figure S1: Predicted versus expected values for**
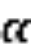
 **in Lahore (green) and all other districts (red) for the environmental model.**

**Additional information about collection of epidemiological data.**

Secondary data from hospital records were used from Punjab province in Pakistan. The data was initially collected by Punjab Health Department as part of the dengue prevention and eradication program. For ensuring accurate reporting from the health facilities, Punjab Health Department used the following three procedures: (i) Clinical case reporting, (ii) Lab case reporting, and (iii) Case management. All health facilities were liable to record the data and share them with Punjab Information Technology Board (PITB) within 24 hours. For clinical case reporting, as per Dengue Expert Advisory Group (DEAG), guidelines, dengue suspects, probable, and confirmed cases needed to be correctly entered on the PITB dashboard within 24 hours of admission. For lab case reporting, all private sector labs must send reports of positive dengue cases in the line list format to the respective Executive District Officer Health (EDOH) for online entry on the dashboard, again within 24 hours. For case management, the healthcare facilities are liable to mange dengue cases regularly in specified Dengue care units, OPDs, emergency units, or wards.

**Video S1:** Video showing the spatial and temporal dynamics of dengue in the study area across Pakistan.
